# An Exclusively Skewed Distribution of Pediatric Immune Reconstitution Inflammatory Syndrome Toward the Female Sex Is Associated With Advanced Acquired Immune Deficiency Syndrome

**DOI:** 10.3389/fped.2019.00293

**Published:** 2019-07-10

**Authors:** Regina Célia de Souza Campos Fernandes, Thaís Louvain de Souza, Thiago da Silva Barcellos, Enrique Medina-Acosta

**Affiliations:** ^1^Faculty of Medicine of Campos, Campos dos Goytacazes, Brazil; ^2^Municipal Program for the Surveillance of Sexually Transmitted Diseases and Acquired Immunodeficiency Syndrome of Campos dos Goytacazes, Campos dos Goytacazes, Brazil; ^3^Molecular Identification and Diagnosis Unit, Laboratory of Biotechnology, Center for Biosciences and Biotechnology, Universidade Estadual do Norte Fluminense Darcy Ribeiro, Campos dos Goytacazes, Brazil

**Keywords:** acquired immune deficiency syndrome (IRIS), antiretroviral therapy (ART), human immunodeficiency virus (HIV), immune reconstitution inflammatory syndrome (IRIS), skewed female sex ratio, X-chromosome inactivation (XCI)

## Abstract

In human immunodeficiency virus and acquired immune deficiency syndrome (HIV/AIDS) patients with very low CD4 cell counts, there is a temporal relationship between administration of antiretroviral therapy (ART) and an increased inflammatory response state known as the immune reconstitution inflammatory syndrome (IRIS). The predominant clinical presentation of IRIS is an infectious disease that can be life-threatening. IRIS-related infectious events are distributed similarly between adult males and females, albeit a few studies have shown a skewing toward the male sex in pediatric IRIS. Here, we assessed sex-specific differences in the causes and extent of IRIS infectious events in HIV-infected pediatric patients on ART. We carried out a prospective clinical analysis (from 2000 to 2018) of IRIS-related infectious events after ART in a cohort of 82 Brazilian children and adolescents infected with HIV-1 through mother-to-child transmission as well as a comprehensive cross-referencing with public records on IRIS-related infectious causes in pediatric HIV/AIDS. Twelve events fulfilling the criteria of IRIS occurred exclusively in 11 females in our cohort. The median age at IRIS events was 3.6 years. The infectious causes included *Mycobacterium bovis*, varicella-zoster virus, molluscum contagiosum virus, human papillomavirus, cytomegalovirus, and *Mycobacterium tuberculosis*. In one female, there was regional bacillus Calmette-Guérin dissemination and cytomegalovirus esophagitis. There was complete health recovery after 10 IRIS events without the use of corticosteroids or ART interruption. One case of IRIS-associated miliary tuberculosis was fatal. The biological female sex was a significant risk factor for IRIS events (odds ratio: 23.67; 95% confidence interval 95%: 1.341–417.7; *P* = 0.0016 and *P* < 0.01 by the multivariable analysis). We observed an effect of the advanced HIV/AIDS variable in IRIS females as compared with non-IRIS females (mean CD4^+^ T cell percentage 13.36 vs. 18.63%; *P* = 0.0489 and *P* < 0.05 by the multivariable analysis), underpinning the exclusively skewed distribution toward the female sex of this cohort. Moreover, the IRIS females in our cohort had higher mean CD4^+^ T cell percentages before (13.36%) and after IRIS (26.56%) than those of the IRIS females (before IRIS, 4.978%; after IRIS, 13.81%) in previous studies conducted worldwide. The exclusively skewed distribution of pediatric IRIS toward the female sex in the cohort was not linked to preferential X-chromosome inactivation rates. We concluded that the exclusively skewed distribution of pediatric IRIS toward females is associated with more advanced AIDS.

## Introduction

Rigorous adherence to antiretroviral therapy (ART) leads to recovery from immunodeficiency and results in a rapid decrease in morbidity and mortality rates among human immunodeficiency virus (HIV)-1 infected patients. In ART patients with very low CD4 cell percentages (CD4%), there is a temporal relationship between therapy and an increased inflammatory response state known as the immune reconstitution inflammatory syndrome (IRIS), occurring a few weeks to months after therapy administration. The onset of IRIS involves clinical manifestations that can be life-threatening and coincides with an elevation in CD4% and a drop in HIV-1 loads (1–3). In most cases, IRIS manifests as opportunistic infections. IRIS-related infectious events can be classified as unmasking, in which there is a subclinical and therefore unrecognized infection that is unveiled after ART, or paradoxical, in which there is an exacerbation of an infectious disease previously observed in the patients (3–5).

The most common infectious agents associated with IRIS manifestations are tuberculosis (TB) or non-tuberculous mycobacteria, cryptococci, herpesvirus, cytomegalovirus (CMV), hepatitis B, and C viruses, John Cunningham virus, and Pneumocystis spp. (1). In severely immunocompromised HIV-infected adults, the onset of IRIS-related TB ranges from 10 to 14 days after ART (6). In pediatric HIV/AIDS, IRIS-related TB can occur up to 6 months after ART (7–14). Grave's autoimmune disease as a manifestation of late-onset IRIS (>12 months) has been reported in adults (15), but in only one pediatric patient (16). When corticosteroid treatment of IRIS-associated infections is ineffective, and life is threatened (17), ART interruption must be considered (18).

IRIS-related infectious events remain a challenge in the management of HIV-infected pediatric patients. Notwithstanding the scarcity of studies, the incidence of IRIS in children on ART ranges from 4.7 to 38% (19, 20) and the associated risk factors vary from one study to another. For example, in one study on 162 pediatric patients from Uganda, male sex, pretreatment low CD4%, CD8 cell count, and coughing were determined IRIS risk factors (19). In a second study on 494 pediatric patients from South Africa (20), increased risk for IRIS was associated with age <12 months. While the viral loads in the IRIS groups are significantly higher than in the non-IRIS groups (11, 21), a lower CD4% in subjects presenting with IRIS-related infectious events is a risk factor (21, 22).

Although a statistically significant association between a biased male/female sex ratio and IRIS has not described (18), a trend toward the male sex has been reported (19). Herein, we assessed sex-specific differences in the causes and extent of IRIS-related infectious events in HIV-infected Brazilian pediatric patients on ART.

## Materials and Methods

### Study Design and Experimental Setting

Prospective observational study on children and adolescents with HIV infection. Subjects were enrolled from August 2000 to August 2018 in the Specialized Assistance Service of the Municipal Program for the Surveillance of Sexually Transmitted Diseases and AIDS of the city of Campos dos Goytacazes (population 463,731; 2010 census), Rio de Janeiro, Brazil. Clinical examination was performed by only one infectious disease specialist pediatrician (RF). The follow-up of male and female subjects was performed monthly or whenever the clinical condition demanded without bias toward either sex. The inclusion criteria were: Subjects under 18 years of age with confirmed HIV infection by mother-to-child transmission and who were on ART (*n* = 82). The cohort includes two cases of IRIS-associated BCGitis, whose clinical presentations were previously reported (23, 24). We adhered to their classification as unmaking and paradoxical IRIS according to the recommendations by French (3), Rabie et al. (4), and French (5). Unmasking IRIS refers to a subclinical and therefore underlying occult infection that is unveiled by the immune response after ART. Paradoxical IRIS is an exacerbation of an infectious disease previously observed in the patients.

### Ethical Considerations

The study received approval (FR-405294) from the Regional Committee of Ethics in Research in Humans from the Faculty of Medicine of Campos. All legally authorized next-of-kin gave written informed consent on behalf of participants in compliance with the Declaration of Helsinki.

### Antiretroviral Therapy

ART was provided universally for infants with confirmed HIV infection during the first year of life and for children or adolescents with moderate or severe clinical manifestations or immunodepression (CD4% <25%), following the recommendations of the Brazilian Ministry of Health (25). It is of note that during the 18 years of the study, different ART regimens were implemented according to the national treatment guidelines.

### Definition Criteria of IRIS

We used the major and minor criteria for IRIS listed by French and colleagues (1); cases required compliance with the two major criteria or one major criterion plus two minor criteria for inclusion. The major criteria are (i) atypical presentation of opportunistic infections or tumors in patients responding to ART and (ii) decrease in plasma HIV RNA concentration by > 1 log copies/mL. The minor criteria are (i) increase in blood CD4% after ART, (ii) increase in an immune response specific to the relevant pathogen, and (iii) spontaneous resolution of the infectious episode without specific antimicrobial therapy or tumor chemotherapy with the continuation of ART (1).

### Estimates of X-chromosome Inactivation

Genomic DNA samples were extracted from peripheral blood of females with IRIS-related infectious events. The extent of X-chromosome inactivation (XCI) was then estimated by interrogating the 5^me^CpG epigenetic marks neighboring short tandem repeats localized in the promoter regions of either the X-linked retinitis pigmentosa *RP2* gene (Xp region) or the androgen receptor *AR* gene (Xq region), using the assay previously reported by us (26).

### Searching Relevant Cases via PubMed

We carried out a literature review following the Preferred Reporting Items for Systematic Reviews and Meta-Analyses (PRISMA) statement guidelines (27) to extract data regarding IRIS-related events from clinical case reports and case series published in the English language. We searched by relevant biomedical tags in the PubMed database of the National Library of Medicine (https://www.ncbi.nlm.nih.gov/pubmed/) from January 1st, 1979 through August 30th, 2018 using the EndNote X9 (Clarivate Analytics, Philadelphia, PA) reference managing software. The following terms were used in pairs, IRIS, HIV, child, children, immunodeficiency, and infant. To be manually reviewed by two annotators and listed as relevant semantic context, a clinical case was required to be a child or adolescent (age <18 years old), has at least a positive HIV-1 serologic test, and an opportunistic infection with atypical presentation after ART introduction.

### Data Analysis

We used the EpiInfo^TM^ public suite from the Centers for Disease Control and Prevention, USA (28) to analyze data variables and to statistically evaluate possible associations between risk factors and the observed IRIS outcome. Multivariable analysis was conducted using logistic regression in the R software package (29) and variables were included in the final model when associated with the outcome (IRIS-related infectious event) with significance *P* < 0.05.

## Results

Throughout 18 years, we prospectively studied 82 HIV-infected children and adolescents on ART (36 males and 46 females). Two patients, male and female, were lost to specialist follow-up because they moved to another city or country. In the remainder, no males presented with IRIS, while 11 (13.8%) females developed 12 IRIS-related infectious events [risk ratio [RR]: 23.67; 95% confidence interval [CI]: 1.341–417.7; *P* = 0.0016 and *P* < 0.01 by the multivariable analysis]. The median time to first symptom presentation following ART was 60 days (mean 108.6 ± 42.41 days). Clinical manifestations, laboratory findings, therapeutic approaches, and evolution of patients with IRIS-related infectious events are summarized in Table 1. For subjects who did not present with IRIS-related events, laboratory findings are listed in Supplementary Table S1.

**Table 1:** Characteristics of clinical manifestations and staging, laboratory findings, therapeutic approach, and evolution of female patients with IRIS.

	**Characteristics of HIV-infection**						**Characteristics of IRIS events**				
**Patient**	**ART regimen^**a**^**	**Stage**	**CD4** (**%**)		**Viral load** (**copies/mL**)		**Age at IRIS**	**Year of event**	**ART timing^**b**^**	**Clinical manifestation**	**Evolution**
			**Basal**	**IRIS**	**Basal**	**IRIS**					
1	DDI, 3TC, EFV, LPV/Rt	A3	2.4	25.7	19,000	1,400	12 years	2003	6 months	Genital HPV	Complete recovery
2	AZT, DDI, NVP	A3	9.6	30.4	110,000	990	4 years	2004	3 months	Molluscum contagiosum	Complete recovery
3	AZT, 3TC, LPV/Rt	C3	13.3	29.7	5,130,000	1,410	8 months	2006	41 days	Regional BCG dissemination	Complete recovery
4	AZT, 3TC, LPV/Rt	C1	27.2	30	1,200,000	11,400	7 months	2006	19 days	Regional BCG dissemination	Complete recovery
4	AZT, 3TC, LPV/Rt	C1	27.2	30	1,200,000	11,400	7 months	2006	2 months	Esophagus disease by CMV	Distal esophageal stenosis
5	AZT, 3TC, EFV	A3	9	20	137,373	689	7 years	2008	20 days	Herpes zoster	Complete recovery
6	AZT, 3TC, EFV	C3	9.9	21	62,300	undetected	3 years	2008	9 months	Perianal HPV	Complete recovery
7	AZT, 3TC, EFV	C3	13.47	24.45	334,545	803	21 months	2010	2 months	Molluscum contagiosum	Complete recovery
8	TDF, 3TC, EFV, DRV, Rt	C3	19.79	25.49	20,272	undetected	18 years	2011	4 months	Herpes zoster	Complete recovery
9	AZT, 3TC, EFV	A3	8.2	8.97	>500,000	937	9 years	2012	5 months	Herpes zoster	Complete recovery
10	TDF, 3TC, LPV/Rt	C3	14.29	–	1,012,485	–	16 years	2014	14 days	Miliary tuberculosis	death
11	AZT, 3TC, LPV/Rt	C3	6.01	46.40	48,519	4,473	10 years	2016	9 days	Tuberculous pneumonia	Complete recovery

a *DDI, Didanosine; 3TC, Lamivudine; EFV, Efavirenz; LPV, Lopinavir; Rt, Ritonavir; NVP, Nevirapine; DRV, Darunavir; TDF, Tenofovir*.

b *Time to onset of IRIS event after ART*.

### Clinical Summary

There were two *Mycobacterium bovis* bacillus Calmette-Guérin (BCG)-related IRIS infectious events, occurring 41 and 18 days after ART introduction; they were treated with isoniazid (10 mg/kg/day), and ART was maintained. Surgical manipulation of lesions was contraindicated. Varicella-zoster virus occurred in three cases of the dermatomal disease with excellent response to acyclovir therapy. Human papillomavirus infection was implicated in two IRIS cases. In a girl with poor adherence to ART, one event of HPV infection was reported 18 months after Zidovudine and Lamivudine treatment, following the Brazilian Guidelines at the time. Nine months later, Efavirenz was added, with perianal HPV infection presenting after a further 9 months.

Two more IRIS cases were caused by a molluscipox infection in the thoracic region with good evolution. One infant female presented with ulcerations at the posterior palate 2 weeks before ART that was managed with acyclovir; she developed vomiting and feeding intolerance and was diagnosed with an esophageal stricture. CMV exposure was confirmed by serology at 12 months [immunoglobulin [Ig] G: 1070.7 UA/mL; IgM: 0.86 UA/mL]. Exteriorization of the proximal esophagus, gastrostomy, and dilatation were performed, and in 2017, her digestive tract was successfully reconstructed. An adolescent female, without adequate adherence to ART and with pulmonary TB that was treated earlier, presented with weight loss, cervical adenopathy, respiratory distress, and miliary radiological pattern after 14 days of supervised ART, which progressed to death in 3 days. Lastly, a 10-year-old female was diagnosed with bacterial pneumonia 9 days after commencing ART. At home, she was treated with penicillin, and developed fever, respiratory distress, weight loss, and bilateral lung infiltrates after 60 days, requiring hospitalization. She had a negative tuberculin test result, albeit household TB contact was reported. She was then treated with rifampicin, isoniazid, and pyrazinamide with complete recovery.

**Figure 1:**
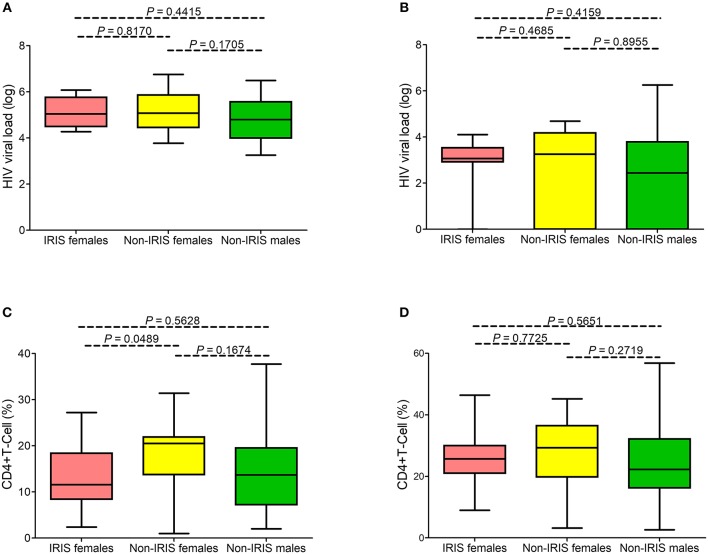
Virologic and immunology profiles in the Brazilian pediatric HIV cohort. No statistical difference was observed between viral loads before **(A)** and after ART **(B)** in females presenting with IRIS-related infectious events (represented in pink), females with no IRIS events (yellow), and males (green). Females presenting with IRIS infectious events exhibited lower CD4 cell percentages before **(C)** but not after ART **(D)**.

In total, we observed 11 episodes of unmasked IRIS and one of paradoxical IRIS. When comparing viral loads (Figures 1A,B) and CD4% (Figures 1C,D), females with IRIS had significantly (*P* = 0.0489 and *P* < 0.05 by the multivariable analysis) lower CD4 values than those of non-IRIS females and viral loads were similar to those of non-IRIS females and males (Figure 1).

The exclusively skewed occurrence of IRIS-related infectious events toward the female sex in a cohort of patients is intriguing. Males and females are known to exhibit contrasting susceptibilities to infectious and non-infectious inflammatory diseases (30). The differential susceptibility to infectious diseases between males and females has been linked to a sex-specific innate immune inflammatory response (31). Moreover, preferential X-chromosome inactivation (XCI) toward one X chromosome in females has been implicated in several X-linked human diseases, including susceptibility to infectious agents (32), and X-linked primary immunodeficiencies (33). Highly skewed XCI is not rare in the general female population (34, 35). Therefore, we investigated a possible association between extremely skewed female sex distribution of IRIS manifestations and affected females exhibiting highly skewed XCI. We only had genomic DNA samples available from six female patients for the XCI assay. Notably, only one affected female exhibited a preferential (>90%) XCI (Supplementary Figure S1).

### IRIS-related Infectious Events in Pediatric HIV/AIDS Reported in the Literature

The literature search yielded 169 publications, but only 40 studies passed our requirements for listing relevant cases (Supplementary Table S2). In total, we listed 127 HIV-1 infected children and adolescents with IRIS-related infectious events. Most cases (80.1%) were from Peru, South Africa, and Thailand. IRIS-related events were distributed between males (37.8%) and females (48%); gender was not indicated for 18 reported cases. The age at IRIS-related infectious events exhibited a bimodal distribution with 28.3% (36/127) of cases occurring before age 1-year and 32.3% (41/127) between the ages of 8- to 11-year. The period from commencing ART to IRIS manifestation ranged from 0 to 120 days, with 54.3% (69/127) of cases occurring 6 weeks after ART. Fatal outcomes after IRIS-related disease occurred in 12.6% (16/127) of cases.

Out of all IRIS-related events, 14.2% (18/127) were pulmonary tuberculosis (Supplementary Table S2), 7.0% (9/127) non-tuberculous mycobacterial disease, and 22.8% (29/127) BCG vaccine adverse events. Neurological disorders like progressive multifocal leukoencephalitis and cryptococcal meningitis occurred in 2.4% (3/127) and 3.1% (4/127) of the cases, respectively.

**Figure 2:**
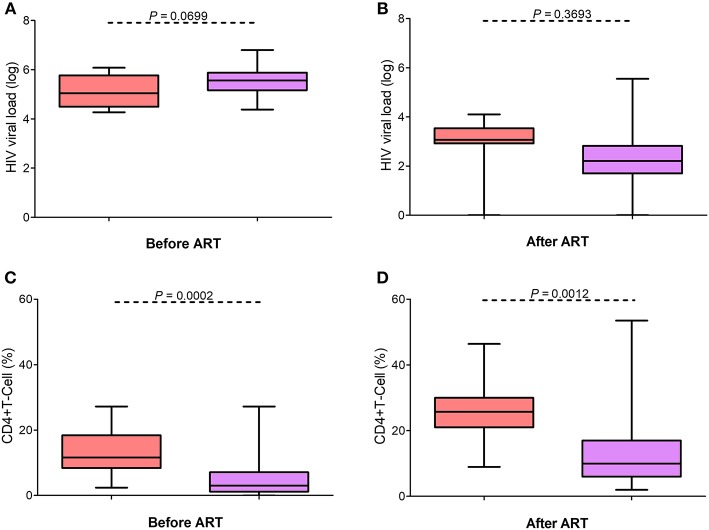
Cross-referencing virologic and immunology profiles in IRIS patients with published reports. No statistical difference was observed between the viral loads in our cohort (pink) and the cases reported in the literature compilation (represented in lilac) at baseline **(A)** and after ART **(B)**. Females presenting with IRIS infectious events in the literature compilation presented lower CD4+ T-cell percentages before **(C)** and after ART **(D)** than the females with IRIS in our cohort.

Viral load >1,000 copies (>3 log) before IRIS events were observed in 84.9% (79/93) of cases. After the onset of IRIS-related infection events, viral loads <1000 (<3 log) were reported in 70.6% (60/85) of cases, with 44.9% presenting with >1,000 (>3 log); in 11.8% (10/85) of cases, the viral load was undetectable. When restricting the analysis to female subsets from the compilation and our cohort, the viral loads were found decreased in both subgroups (Figures 2A,B).

Before the onset of IRIS, 85.7% (96/112) of the reported cases had CD4 cell counts <200 cells/mL (<15%). After IRIS, CD4 cell counts increased to 200–499 (>15%) in 40.4% (40/99) of the cases. When the female subsets were compared, we noted that the compilation subset had significantly lower CD4 counts (*P* = 0.0002) than that of our cohort and that there was an increase in CD4% in both groups (Figures 2C,D). The IRIS females of our cohort had higher mean CD4^+^ T cell percentages before (13.36%) and after IRIS (26.56%) than those of the IRIS females (before IRIS, 4.9%; after IRIS, 13.8%) in previous studies conducted worldwide. Interestingly, the IRIS females in the literature have more advanced HIV/AIDS than the IRIS females of our cohort.

## Discussion

We identified an abnormal distribution, fully skewed toward the female sex, of IRIS-related infectious events in HIV-infected Brazilian children and adolescents on ART. We observed 12 episodes in 11 females and none in males during a study period of 18 years. The bulk of clinical data allowed us to suggest that the observed skewed distribution toward the female sex is due to more advanced HIV/AIDS in these females than in non-IRIS females. Cross-referencing with pediatric HIV/AIDS data from the literature revealed a fair distribution of reported IRIS-related infectious events between females and males (11, 18, 20, 22), except for a study in Uganda that reported a bias toward the male sex (*P* = 0.010) (19). In adults with HIV/AIDS, IRIS-related infectious events occurred at the same rate in both females and males (36–39), albeit one study reported a male bias [*P* = 0.018; (40)]. The IRIS females in our cohort had higher mean CD4^+^ T cell percentages before and after IRIS than those of the IRIS females in previous studies conducted worldwide.

Sex-biased susceptibility to bacterial infections has been linked to the differential effects of sex steroid hormones (estrogen and testosterone) on innate immunity (41); for example, males are more susceptible to TB than females (42). A caveat against sex hormones being involved in the female-biased presentation of IRIS in our cohort is the fact that none of the males presented with IRIS. Unfortunately, we did not measure sex hormones in the 11 affected females at the time of IRIS events, and it is thus unclear whether abnormal sex hormone levels are implicated in female-biased IRIS manifestations. We did investigate, however, whether this skewed IRIS event distribution toward females was associated with highly skewed XCI. A significant number of immune-associated genes map to the X-chromosome (43) and preferential XCI in females can result in phenotypic susceptibility and disease. In female eutherian mammals with normal XCI, the epigenetic transcriptional silencing of an X-chromosome in each somatic cell occurs at random during the early stages of embryonic development, assuring monoallelic expression in each cell and compensating for dosage-sensitive X-linked genes between females (XX) and males (XY) (26). Although we only had genomic DNA samples from six female patients, the complete skewing toward the female sex could not be explained by discrete differences in the rates of XCI tested in blood.

Our study exemplified the broad spectrum of etiological agents associated with IRIS-related infectious events in childhood and adolescence. IRIS-related BCG regional adenitis occurred in 16.6% (2/12) of cases, highlighting the breadth of association between HIV infection and BCG vaccination at birth (4, 23, 44). Esophageal strictures infrequently complicate the presentation of CMV disease in HIV-infected adults (45) and there appears to be a functional association between IRIS and esophageal stricture observed in CMV infection cases (45). To our knowledge, we report the first pediatric case of esophageal stricture secondary to CMV infection related to IRIS; moreover, CD4% during the IRIS event was >25, corroborating the view that CD4% is not a reliable marker for disease progression and severity in infants.

IRIS-related infectious events should be considered important contributors to higher mortality rates in resource-limited settings with a late introduction of ART (46). Very early ART administration is an essential preventive factor against an IRIS-related fatal outcome (4). Furthermore, IRIS-related infectious events are more life-threatening at an early age (21). In our cohort, six IRIS-related episodes occurred at <1year of age. None of the 11 females were treated with corticosteroids, and all remained on ART. Recovery was completed in 10 females, but there was a fatal case for a 16-year-old patient (mortality of 8.3%).

The prevalence of reported IRIS-related infectious events varied significantly by country or geographical region; 4.7% of the cases were reported in South Africa (20), 5.9% in the United Kingdom (47), 11.5–16.4% in the USA (48, 49), 18.9% in India (18), 19% in Thailand (22), 20% in Peru (11), 38.3% in Uganda (19), 23% in Latin America (50), and 22% in Malawi and Botswana (51). The lower rates may in part be explained by socio-economic status (i.e., better nutritional status of patients), moderate manifestations of HIV infection, and varying compliances with the criteria of the definition. Importantly, many of the cases are from Africa, where the presentation to care is often late, which may help also explaining why this phenomenon is noted. It is unclear whether differential (epi)genetic components can partly account for the disparity in distribution. Sex-stratified genome-wide association studies of IRIS using multiethnic genotyping arrays are needed to appraise the differences in disease susceptibility and to identify candidate autosomal and X-linked loci in diverse and admixed populations.

In conclusion, our prospective study found that IRIS-related infectious events occurred exclusively in females in a cohort of 80 HIV-infected Brazilian children and adolescents on ART. This complete skewing toward the female sex is uncommon and was linked to more advanced HIV/AIDS. The findings presented here should be interpreted with caution, because the following limitations: first, albeit being a prospective cohort study, we cannot rule out recall bias. Second, the sample number is small and, third, the study was restricted to one surveillance service. We expect that similar observational studies replicate our intriguing findings.

## Ethics Statement

The study received approval (FR-405294) from the Regional Committee of Ethics in Research in Humans from the Faculty of Medicine of Campos. All legally authorized next-of-kin gave written informed consent on behalf of participants in compliance with the Declaration of Helsinki.

## Author Contributions

Rds, TL, and EM-A designed the study, analyzed data, wrote, and edited the typescript. Rds performed the clinical follow-up. TL performed XCI assays. TL and TdS carried out the literature review. All the authors gave final approval.

### Conflict of Interest Statement

The authors declare that the research was conducted in the absence of any commercial or financial relationships that could be construed as a potential conflict of interest.

## Supplementary Material

Supplementary Figure S1 Rates of X-chromosome inactivation (XCI) in six females presenting with IRIS-related infectious events. The percentage of XCI was determined by genotyping genomic DNA with the two highly polymorphic short tandem repeat loci located in the *AR* and *RP2* genes (26) to identify heterozygotes. Both *AR* and *RP2* genes escape XCI and thus in females with random XCI, the rate of X-chromosome active (Xa) over X-chromosome inactive (Xi) is ~50%. The figure depicts the Xa/Xi rate (in percentage) observed for the *AR* and *RP2* marker systems. Only one female sample exhibited a highly skewed rate of XCI (>90%).

Supplementary Table S1 Clinical staging, classification, and monitoring characteristics for HIV/AIDS pediatric patients who did not present with IRIS-related infectious events in our cohort.

Supplementary Table S2 PubMed-based compilation of IRIS-related infectious events in children and adolescents infected with HIV (January 1st, 1979 through August 30th, 2018).
