# Supplementary figures and images for: An Exclusively Skewed Distribution of Pediatric Immune Reconstitution Inflammatory Syndrome Toward the Female Sex Is Associated With Advanced Acquired Immune Deficiency Syndrome
Source: Front Pediatr. 2019 Jul 10;7:293. doi: 10.3389/fped.2019.00293 (PMC6635464; doi:10.3389/fped.2019.00293)

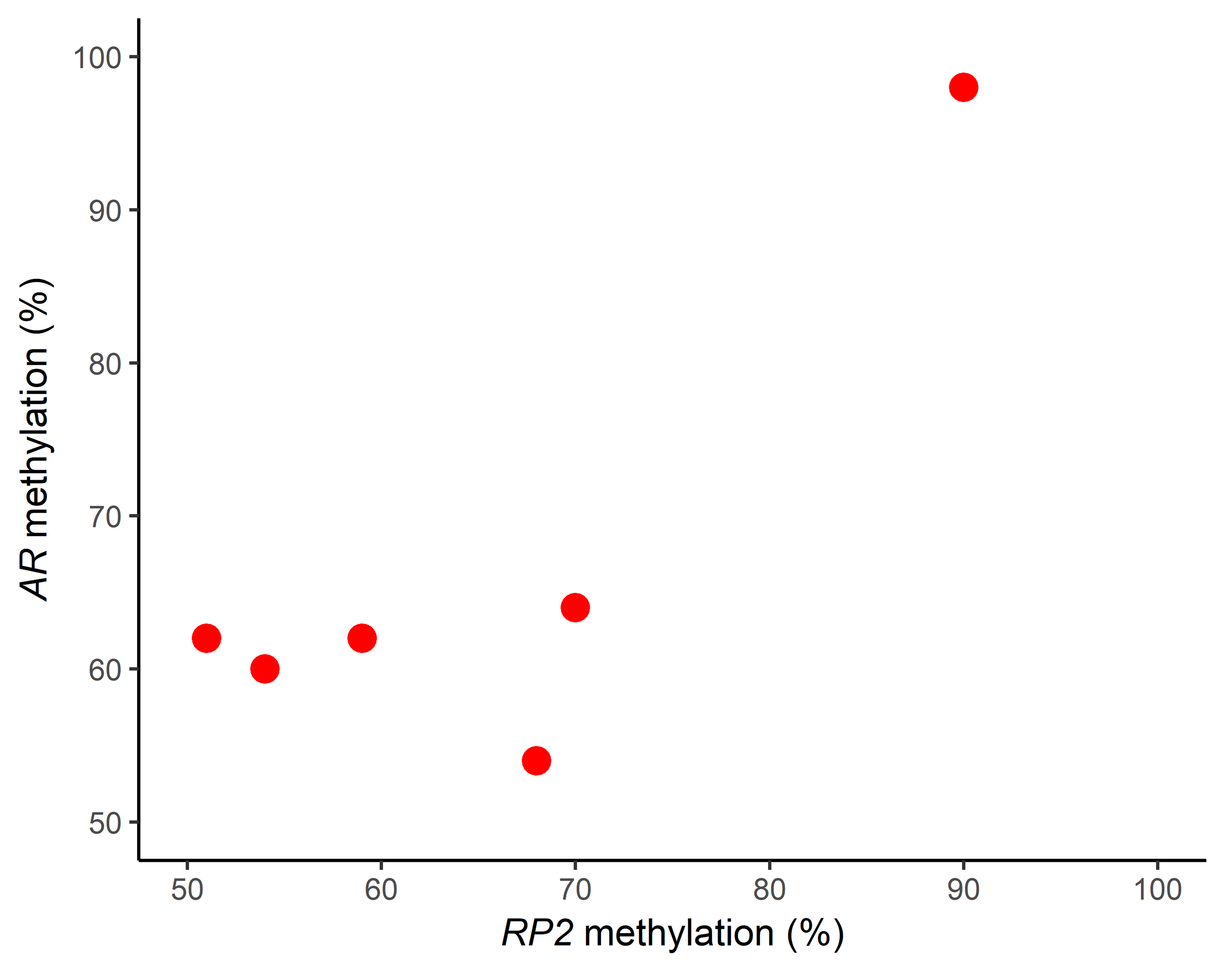

Supplement: Supplementary Figure S1 — Rates of X-chromosome inactivation (XCI) in six females presenting with IRIS-related infectious events. The percentage of XCI was determined by genotyping genomic DNA with the two highly polymorphic short tandem repeat loci located in the AR and RP2 genes (26) to identify heterozygotes. Both AR and RP2 genes escape XCI and thus in females with random XCI, the rate of X-chromosome active (Xa) over X-chromosome inactive (Xi) is ~50%. The figure depicts the Xa/Xi rate (in percentage) observed for the AR and RP2 marker systems. Only one female sample exhibited a highly skewed rate of XCI (>90%). [file Image_1.TIFF]
